# Does induction of labor for constitutionally large-for-gestational-age fetuses identified in utero reduce maternal morbidity?

**DOI:** 10.1186/1471-2393-14-156

**Published:** 2014-05-01

**Authors:** Françoise Vendittelli, Olivier Rivière, Brigitte Neveu, Didier Lémery

**Affiliations:** 1 Faculté de médecine RTH Laennec, The AUDIPOG Sentinel Network (Association des Utilisateurs de Dossiers informatisés en Pédiatrie, Obstétrique et Gynécologie), 7 Rue guillaume Paradin, 69372 Lyon Cedex 08, France; 2 Centre Hospitalo-Universitaire de Clermont-Ferrand, Site Estaing, Pôle de Gynécologie-Obstétrique et Biologie de la Reproduction Humaine, Place Lucie et Raymond Aubrac, 63003 Clermont-Ferrand Cedex 1, France; 3 Clermont Université, Université d’Auvergne, EA 4681, PEPRADE (Périnatalité, grossesse, Environnement, PRAtiques médicales et DEveloppement), CHU de Clermont-Ferrand, Site Estaing, 1 place Lucie et Raymond Aubrac, 63003 Clermont-Ferrand Cedex 1, France; 4 Institut Mutualiste Montsouris, 40 Boulevard Jourdan, 75674 Paris Cedex 14, France

**Keywords:** Cesarean, Delivery, Episiotomy, Fetal macrosomia, Induced labor, Large-for-gestational-age, Maternal morbidity, Neonatal morbidity, Perineal tears

## Abstract

**Background:**

The number of infants with a birth weight > 97^th^ percentile for gestational age has increased over the years. Although some studies have examined the interest of inducing labor for fetuses with macrosomia suspected in utero, only a few have analyzed this suspected macrosomia according to estimated weight at each gestational age. Most studies have focused principally on neonatal rather than on maternal (and still less on perineal) outcomes. The principal aim of this study was to assess whether a policy of induction of labor for women with a constitutionally large-for-gestational-age fetus might reduce the occurrence of severe perineal tears; the secondary aims of this work were to assess whether this policy would reduce either recourse to cesarean delivery during labor or neonatal complications.

**Methods:**

This historical cohort study (n = 3077) analyzed records from a French perinatal database. Women without diabetes and with a cephalic singleton term pregnancy were eligible for the study. We excluded medically indicated terminations of pregnancy and in utero fetal deaths. Among the pregnancies with fetuses suspected, before birth, of being large-for-gestational-age, we compared those for whom labor was induced from ≥ 37 weeks to ≤ 38 weeks^+ 6 days^ (n = 199) to those with expectant obstetrical management (n = 2878). In this intention-to-treat analysis, results were expressed as crude and adjusted relative risks.

**Results:**

The mean birth weight was 4012 g ± 421 g. The rate of perineal lesions did not differ between the two groups in either primiparas (aRR: 1.06; 95% CI: 0.86-1.31) or multiparas (aRR: 0.94; 95% CI: 0.84-1.05). Similarly, neither the cesarean rate (aRR: 1.11; 95% CI: 0.82-1.50) nor the risks of resuscitation in the delivery room or of death in the delivery room or in the immediate postpartum or of neonatal transfer to the NICU (aRR = 0.94; 95% CI: 0.59-1.50) differed between the two groups.

**Conclusions:**

A policy of induction of labor for women with a constitutionally large-for-gestational-age fetus among women without diabetes does not reduce maternal morbidity.

## Background

The number of infants with a birth weight > 97^th^ percentile for gestational age has increased over the years. Accordingly, in France, 2.3% children had a birth weight > 97^th^ percentile at birth in 1994–1996 and 2.5% in 2006–2008 [1]. The percentage of cesarean deliveries in this group also increased over the same period — from 8.2% to 14.4% before labor and from 11.8% to 15.8% during labor [2].

The episiotomy rate is also a concern in France, as in other countries; it has fallen from 56.0% in 1994 to 35.8% in 2008 [1,3]. During the same period, severe perineal lesions (3^th^ and 4^th^ degree) remained stable in France (around 0.9%). In 2005, the French College of Gynecologists and Obstetricians (CNGOF) issued clinical practice guidelines to reduce this rate [3]. Numerous publications have demonstrated that perineal lacerations during delivery, especially severe lacerations, are often associated with an elevated birth weight [4-6].

The purpose of prenatal screening for macrosomia is to limit its neonatal and maternal consequences. Studies have looked at the mode of delivery and especially the potential advantage of elective cesarean deliveries [7-9]. Most studies, however, have examined the increase in intrapartum, neonatal, and maternal complications associated with the birth of a macrosomic neonate, defined as a child with a birth weight exceeding 4000 g, regardless of gestational age [10-15]. Studies examining the utility of inducing labor have generally used a retrospective analysis based on actual birth weight, rather than estimated fetal weight before birth [16,17]. Some studies have examined the benefits of inducing labor for fetuses with macrosomia suspected in utero [18-23]. Among the latter, only a few analyzed this suspected macrosomia according to estimated weight at each gestational age [20]. A meta-analysis including 3 randomized clinical trials involving 372 women did not find that induction affected the risk of maternal or neonatal morbidity, but the power of these studies to show a difference in rare events was limited [18]. They focused especially on neonatal rather than on maternal (and still less on perineal) outcomes [19,20,22,23].

Knowing that a policy restricting the use of episiotomy compared with routine episiotomy is favored [24], we sought to assess whether a policy of induction of labor for women with in utero identification of constitutionally large-for-gestational-age fetuses (LGA) might reduce the occurrence of perineal tears. The secondary objectives were to determine if such a policy reduced either recourse to cesarean delivery during labor or early neonatal complications.

## Methods

### Materials

This historical cohort study concerned all deliveries included in the AUDIPOG sentinel network database. This network, created in 1994, comprises public and private maternity units from every region in France; they contribute individual data on mothers and infants for pooling and analysis. Earlier publications have described its objectives and the database [25,26]. At the time of this study, the database included 411,734 pregnancies from 1994 through 2008 from 233 participating maternity units.

This study was approved by the French institutional review board [IRB 5921 (CECIC) for Rhône-Alpes-Auvergne (Grenoble) in December 2012].

### Inclusion and exclusion criteria

**Figure 1:**
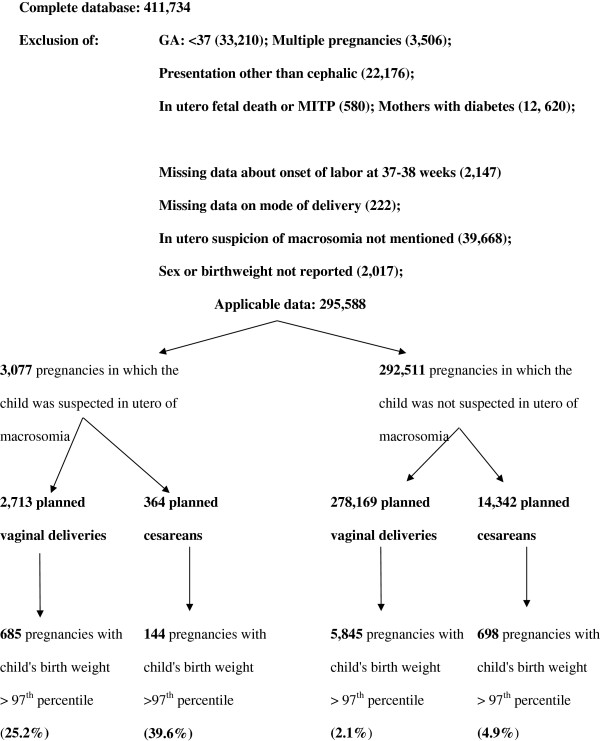
Description of the selection of patients for the study.

Within this cohort we excluded all medical terminations of pregnancy and in utero fetal deaths (n = 580). The study also excluded deliveries before 37 weeks (n = 33,210 women), multiple pregnancies (n = 3506 women), fetal presentations other than cephalic (n = 22,176 pregnancies), women with pregnancy-related or preexisting diabetes (n = 12,620 women), cases for which mode of onset of labor at 37 or 38 weeks was not reported (n = 2147 women), and the files for which information about mode of delivery was missing (n = 222 women). The files containing no mention of estimated fetal weight in utero were excluded (n = 39,668 files). Files where birth weight or sex was missing were also excluded (2017 files). There were therefore 3077 pregnancies in which the fetus was identified with suspected LGA before birth and 292,511 pregnancies for which the fetus was not (Figure 1). The term “LGA” at birth describes a neonate whose birth weight is at least 1.88 standard deviations (SD) higher than the mean (≥2 SD) for the infant’s gestational age and sex, that is, the 97^th^ percentile for gestational age, based on data derived from the reference population included in our database [27]. Specifically, at 37 weeks, LGA was defined as 3836 g for a boy and 3691 g for a girl. Our study includes only cases in which suspected macrosomia was reported in the file during (that is, before the end of) pregnancy and thus before the baby’s birth weight was known.

### Definition of variables and statistical analysis

Among the fetuses suspected of LGA status before birth (defined by French Audipog physicians as > 97^th^ percentile for gestational age), we compared those whose mothers’ labor was induced between ≥ 37 weeks and ≤ 38 weeks^+ 6 days^ (n = 199) to those with expectant obstetrical management during that time period (thus considered the unexposed or control group)(n = 2878). Women remained in the control group if they underwent induction of labor afterwards, that is, at or after 39 weeks, due to the onset of either a maternal or fetal disease in this late stage of pregnancy, or induction for term, either by oxytocin ≥ 41 weeks^+ 0 days^ if the cervix was favorable, or by E2 prostaglandin (at 41 weeks^+ 6 days^), in accordance with standard French practices.

The prenatal diagnosis of cases of suspected LGA in our database was based on the last obstetrical ultrasound examination, during which antenatal fetal weight is estimated between 30 and 35 weeks. In accordance with the national guidelines [28], health insurance in France routinely covers this third-trimester ultrasound examination, which among other things enables fetal weight to be estimated according to French growth curves [27]. In practice, in France, when a fetus is suspected of LGA status, a fourth ultrasound is performed slightly before 37 weeks to confirm this status and plan the mode of delivery.

The principal outcome was perineal lesions, including episiotomy. The secondary outcome measures included the cesarean rate during labor or induction and the rate of severe postpartum hemorrhage (>1 L). Moreover, the secondary outcome measure concerning the infant was a composite criterion defined as a “child who was resuscitated or died in the delivery room or in the immediate postpartum period or was transferred to neonatal intensive care (NICU)”. Other secondary outcome measures were traumatic neonatal lesions (defined as fractures of the clavicle or brachial plexus, skull or facial injuries, facial paralysis, cephalohematoma, or scalp lesions), and low 5-min Apgar scores (≤4 and < 7).

Categorical variables were compared by chi-square tests (or Fisher’s exact test, as appropriate) and continuous variables by Student’s t test. Crude relative risks (RR) of neonatal and maternal complications for macrosomia identified prenatally were calculated, with their 95% confidence intervals (95% CI). A log-binomial model was used to adjust for covariables previously reported in the literature to be either a risk factor or confounding factor for each outcome in our work. Unpublished confounding variables tested in the model were also selected in bivariate analyses if p ≤ .20 and if they were found to be clinically significant. Adjusted RRs were calculated with their 95% CIs. Clinically relevant interactions between the induction of labor and other factors were tested. The level of statistical significance was .05. All statistical analyses were performed with SAS software (version 9, SAS Institute, Inc, Cary, NC, 2002–2010).

## Results

Of the 3077 pregnancies in which the child was suspected of LGA status before birth, 2713 had planned vaginal deliveries and 364 planned cesareans. Among these 3077 pregnancies, 26.9% of the children finally had a birth weight ≥ 97^th^ percentile (Figure 1).

The mean maternal age in our historical cohort was 30.0 (±5.3) years and the mean number of previous deliveries 1.0 (±1.2). Overall, 13.5% of the women smoked during pregnancy, and 43.0% had a body mass index (BMI) ≥ 25 (Tables 1 and 2). Perineal lesions were observed in 75.5% of the women in our cohort (1490/1974) and episiotomy in 54.6%. The rate of prelabor cesareans was 11.8% and the rate of cesareans during labor 18.8%. The severe postpartum hemorrhage rate (>1 L) was 4.5% (111/2476). The mean gestational age at delivery was 39.6 (±1.2) weeks. The mean birth weight was 4012 (±421) g. Among the infants, 5.5% underwent resuscitation in the delivery room (168/3040), and 6.0% were transferred to the NICU immediately or secondarily (181/3000). No child died in the delivery room, in the immediate postpartum, or during labor (0/3077), while 6.1% experienced neonatal trauma (133/2178). The 5-min Apgar score was ≤ 7 in 0.6% (19/2964).

**Table 1:** Description of the social and demographic characteristics

**Women with a suspected large for gestational age fetus**	**Overall cohort**	**Induction of labor at 37–38 weeks**^***+ 6 d***^	**Expectant management**^**c**^	**P value**
	**(n = 3077)%**	**(n = 199)%**	**(n = 2878)%**	
**Maternal age**	(n = 3070)	(n = 198)	(n = 2872)	
< 20–34 years	1.6	3.0	1.5	.24
20-34 years	78.6	76.8	78.7	
≥ 35–34 years	19.8	20.2	19.8	
**Family situation**	(n = 1873)	(n = 124)	(n = 1749)	
Single	8.2	7.3	8.3	.85
Lives with partner	30.5	31.4	30.4	
Married	60.3	59.7	60.4	
Other	1.0	1.6	0.9	
**Geographic origin**	(n = 2064)	(n = 129)	(n = 1935)	
France^a^	74.5	76.7	74.3	.67
Southern Europe	3.4	1.6	3.6	
North Africa	10.1	10.1	10.1	
Other	12.0	11.6	12.0	
**Body mass index**^**b**^	(n = 2619)	(n = 167)	(n = 2452)	
< 20	11.6	7.2	11.9	.18
20-24	45.4	46.7	45.3	
≥ 25	43.0	46.1	42.8	

^a^Continental (metropolitan) France. ^b^BMI: Body mass index at beginning of pregnancy. ^c^No induction of labor between 37 and 38 weeks^+ 6 days^.

**Table 2:** Description of women’s medical and obstetric characteristics

**Women with a suspected large for gestational age fetus**	**Overall cohort (n = 3077)% [m ± ET]**	**Induction of labor at 37–38 weeks**^***+ 6 d***^**(n = 199)**	**Expectant management (n = 2878)% [m ± ET] **	**P value**
**Primiparous**	(n = 2982) 39.7	(n = 193) 34.2	(n = 2789) 40.1	.10
**Uterine scar**	(n = 2725) 12.6	(n = 170) 2.9	(n = 2555) 13.2	<.0001
**History of stillbirth or neonatal death**	(n = 1636) 2.9	(n = 120) 3.3	(n = 1516) 2.8	.77
**Smoked during pregnancy**	(n = 2905)	(n = 190) 12.1	(n = 2715) 13.6	.57
**Any pregnancy-related disorder**	(n = 3077) 45.9	(n = 199) 62.8	(n = 2878) 44.7	<.0001
Polyhydramnios	9.0	16.6	8.5	.0002
Hypertension	6.3	14.0	5.7	<.0001
**Labor onset**	(n = 3046)	(n = 199)	(n = 2847)	
Labor induction^a^	24.1	0	25.8^c^	-
Spontaneous labor	57.6	0	61.6	
Elective cesarean	11.8	0	12.6	
**Mean gestational age**^**b**^	(n = 3077) [39.6 ± 1.2]	(n = 199) [37.7 ± 0.5]	(n = 2878) [39.8 ± 1.2]	<.0001
**Years of delivery**	(n = 3077)	(n = 199)	(n = 2878)	
1994-1997	9.4	7.5	9.6	.28
1998-2001	17.4	21.6	17.1	
2002-2005	34.3	35.7	34.2	
2006-2008	38.9	35.2	39.1	

^a^Labor induction ≥ 39 weeks.

^b^Gestational age at delivery.

^c^Distribution of the labor induction term in the expectant management group: 31.0% at 39 weeks; 23.9% at 40 weeks; 40.4% at 41 weeks, and 4.8% at 42 weeks.

Table 1 also describes the social and demographic characteristics for both groups. There was no statistical difference in maternal age, family situation, women’s geographic origin, or BMI (p > .05). Table 2 describes the women’s medical and obstetric characteristics. The groups did not differ significantly for parity, smoking during pregnancy, or history of stillbirths and neonatal deaths (p > .05) (Table 2). The group with inductions between ≥ 37 weeks and ≤ 38 weeks^+ 6 days^ included a higher percentage of women with a pregnancy-related disease, especially polyhydramnios or hypertension (p ≤ .0002). In the control group, 25.8% of the women finally underwent induction after 39 weeks. The overall control group included a higher proportion of women with a previous cesarean (p < .0001), and their babies had a higher mean gestational age at delivery, the latter outcome inherent in the definition of the groups (p < .0001) (Table 2). Table 3 summarizes the labor and delivery data. Distribution of all the characteristics studied differed statistically between the two groups (p < .05), except for duration of labor, rate of operative vaginal delivery, and medical problems during labor. Accordingly, the control group included more cesareans (31.2% vs. 21.6%) (p = .005), and therefore, more spinal anesthesia (p = .0006) than in the exposed group. We also noted a higher mean birth weight in the control group (4028 ± 417 vs. 3792 ± 418 g) (p < 10^−4^) than in the exposed group (Table 3).

**Table 3:** Description of obstetrical data about labor and delivery

**Women with a suspected large for gestational age fetus**	**Overall cohort (n = 3077)% [m ± ET]**	**Induction of labor at 37–38 weeks**^***+ 6 d***^**(n = 199)% [m ± ET]**	**Expectant management(n = 2878)% [m ± ET]**	**P value**
**Mode of delivery**	(n = 3071)	(n = 199)	(n = 2872)	
Spontaneous delivery	52.0	61.3	51.4	<0.0001
Overall cesareans	30.6	21.6	31.2	.005
Operative VD^a^	17.4	17.1	17.4	.90
Instrumental delivery	94.2^b^	96.8	94.0	
Other maneuvers^c^	5.8	3.2	6.0	
**Mode of anesthesia**	(n = 2879)	(n = 194)	(n = 2685)	
Spinal anesthesia	13.7	4.6	14.3	.0006
Epidural anesthesia	68.9	80.4	68.0	
General anesthesia	2.1	2.6	2.1	
Other	1.1	0	1.2	
**Duration of labor**^**d**^	(n = 1465)	(n = 120)	(n = 1345)	
< 2–6 h	39.4	45.8	38.9	.35
2 - 4 h	36.5	30.0	37.0	
4 - 6 h	16.2	17.5	16.1	
≥ 6–4 h	7.9	6.7	8.0	
**Problem during labor**^**e**^	(n = 2910) 34.5	(n = 197) 35.5	(n = 2713) 34.4	.75
**Birth weight**	(n = 3077) [4012 ± 421]	(n = 199) [3792 ± 418]	(n = 2878) [4028 ± 417]	<.0001
< 3000 g	0.8	2.5	0.7	<.0001
3000 – 3499 g	9.4	21.1	8.6	
3500 – 3999 g	37.0	46.8	36.3	
≥ 4000 g	52.8	29.6	54.4	

^a^Operative VD: operative vaginal delivery.

^b^Instrumental deliveries in the global cohort included: 39.1% with forceps, 11.0% with spatulas, 44.1% by vacuum extractions.

^c^Other manoeuvers = maneuver for shoulders dystocia, internal cephalic version, total breech extraction, etc.

^d^From 5 cm to full dilatation.

^e^Problem during labor of any kind [fetal-pelvic disproportion, fetal heart rate anomaly, failure of induction, dynamic dystocia (hypotonic or hypertonic uterine activity or cervical dystocia), dystocia related to an abnormal position or presentation (abnormal position of the fetal head), maternal disease such as fever or hemorrhage.

We identified the following confounding factors for the principal outcome (overall perineal lesions): parity, uterine scar, pregnancy-related disorder, and type of anesthesia. The confounding factors for the secondary outcomes were parity, BMI, uterine scar, and type of anesthesia for episiotomy alone; parity, pregnancy-related disease, and type of anesthesia for perineal tears; parity, BMI, pregnancy-related disorder, and type of anesthesia for cesareans during labor; parity alone for severe postpartum hemorrhage; parity, BMI, pregnancy-related disorder, and type of anesthesia for resuscitation or death in the delivery room or immediate postpartum or neonatal transfer; and a uterine scar, anesthesia, and birth weight for traumatic neonatal lesions.

The adjusted risk of episiotomy did not differ between the two groups: aRR = 0.93 (95% CI: 0.77-1.06) (Table 4). Because of an interaction between parity and induction between 37–38 weeks, the adjusted RRs for global perineal lesions (including episiotomy) were calculated separately for the primiparas and the multiparas. The RRs of perineal lesions for the primiparas and the multiparas, adjusted for uterine scar, pregnancy-related disease, and the prognostic factors of birth weight and operative vaginal delivery, did not differ between the 2 groups (primiparas: aRR = 1.06; 95% CI: 0.86-1.31 and multiparas: aRR = 0.94; 95% CI: 0.84-1.05) (Table 4). The crude risk of a cesarean during labor in the induction group was no higher than in the control group (RR = 1.16; 95% CI: 0.88-1.53). The adjusted risk was 1.11 (95% CI: 0.82-1.50) (Table 4). Severe postpartum hemorrhage (PPH >1 L) did not differ significantly between the two groups (Table 4): aRR = 0.72 (95% CI: 0.27-1.93).

**Table 4:** Maternal complications according to policy of induced labor at term among large-for-gestational-age fetuses

**Women with a suspected large-for-gestational-age fetus**	**Induction of labor between 37 and 38 weeks**^**+6 d**^**(n = 199)%**	**Expectant management (n = 2878)%**	**Crude RR (95% CI)**	**Adjusted RR (95% CI)**
**Perineal lesions**^**g**^	(n = 150)	(n = 1824)		1.06 (0.86-1.31)^a^
	66.0	76.3	0.87 (0.77-0.97)	0.94 (0.84-1.05)^b^
**Episiotomies**	43.8	55.5	0.79 (0.65-0.95)	0.93 (0.77-1.06)^c^
**Perineal tears**	23.0	25.7	0.89 (0.64-1.25)	1.01 (0.72-1.40)^d^
**1**^**st **^**and 2**^**nd **^**degree**^**h**^	20.6	24.2	-	-
**3**^**rd **^**degree**^**i**^	0.8	1.4	-	-
**4**^**th **^**degree**^**j**^	1.6	0.1	-	-
**Cesarean section**	(n = 199)	(n = 2872)		
**Before labor**	0	12.6	-	-
**During labor**	21.6	18.6	1.16 (0.88-1.53)	1.11 (0.82-1.50)^e^
**Severe maternal postpartum hemorrhage (>1 L)**	(n = 168) 2.4	(n = 2308) 4.6	0.51 (0.19-1.38)	0.72 (0.27-1.93)^f^

^a^RR adjusted for uterine scar, pregnancy-related disease other than large for-gestational-age status, operative vaginal delivery, and birth weight (<4000 g vs. ≥ 4000 g). Because of the interaction between induction and parity, the adjusted RR for induction is that for the primiparas.

^b^RR adjusted for uterine scar, pregnancy-related disease other than large-for-gestational-age status, operative vaginal delivery, and birth weight (<4000 g vs. ≥ 4000 g). Because of the interaction between induction and parity, the adjusted RR for induction is that for the multiparas.

^c^RR adjusted for parity (primiparas vs. multiparas), uterine scar, operative vaginal delivery, anesthesia (none vs. epidural analgesia or spinal anesthesia, vs. general anesthesia, vs. other anesthesia) and birth weight (<4000 g vs. ≥ 4000 g).

^d^RR adjusted for parity (primiparas vs. multiparas), pregnancy-related disease other than large-for-gestational-age status, operative vaginal delivery, anesthesia (none vs. epidural analgesia or spinal anesthesia, vs. general anesthesia, vs. other anesthesia) and birth weight (<4000 g vs. ≥ 4000 g).

^e^RR adjusted for parity (primiparas vs. multiparous), uterine scar, BMI (<20 vs 20–24 vs ≥ 25), pregnancy-related disease other than large-for-gestational-age status, anesthesia (general anesthesia vs. epidural analgesia or spinal anesthesia, vs. other anesthesia) and birth weight (<4000 g vs. ≥ 4000 g).

^f^RR adjusted for parity, (primiparas vs. multiparas), uterine scar and birth weight (<4000 g vs. ≥ 4000 g).

^g^Defined as any type of perineal tear and/or episiotomy.

^h^First-degree tears involve damage to vaginal and perineal skin; second-degree tears involve the posterior vaginal wall and the underlying elevator and perineal muscles.

^i^Third-degree tears involve the anal sphincter, with either total or partial damage to the sphincter and fourth-degree tears involve the anal sphincter and tears into the rectal mucosa.

^j^Fourth-degree tears involve the anal sphincter and tears into the rectal mucosa.

Neonatal outcomes are described in Table 5. The crude and adjusted risk of resuscitation in the delivery room or of death in the delivery room or in the immediate postpartum or of neonatal transfer to the NICU did not differ between the 2 groups: RR = 0.86 (95% CI: 0.54-1.37) and aRR = 0.94 (95% CI: 0.59-1.50) (Table 5). Similarly, the groups did not differ for traumatic neonatal lesions: RR = 1.35 (95% CI: 0.75-2.45) and aRR = 1.53 (95% CI: 0.84-2.79) (Table 5). No 5-min Apgar score ≤ 4 or < 7 was observed in the group with labor induced between 37–38 weeks.

**Table 5:** Neonatal morbidity and mortality according to policy of induced labor at term among LGA fetuses

**Fetuses suspected to be large for gestational age**	**Induction of labor between 37 and 38 weeks**^**+6 d**^**(n = 199)%**	**Expectant management (n = 2878)%**	**Crude RR (95% CI)**	**Adjusted RR (95% CI)**
**Resuscitation in delivery room or death in delivery room or immediate postpartum or neonatal transfer**	(n = 199)	(n = 2878)		
	8.5	10.0	0.86 (0.54-1.37)	0.94 (0.59 -1.50)^b^
**Neonatal trauma**^**a**^	(n = 136) 8.1	(n = 2042) 6.0	1.35 (0.75-2.45)	1.53 (0.84-2.79)^c^
**Fractured clavicle**	4.1	3.0		
**Brachial plexus**	2.0	0.3		
**Other**	2.0	2.7		
**5-min Apgar:**	(n = 198)	(n = 2766)		
**≤ 4**	0	0.3	-	-
**< 7**	0	0.7	-	-

^a^Traumatic neonatal lesions = fractures of the clavicle or brachial plexus, skull or facial injuries, facial paralysis, cephalohematoma, scalp lesions.

^b^RR adjusted for uterine scar, pregnancy-related disease other than women with a suspected large-for-gestational-age fetus, cesarean during labor, operative vaginal delivery and birth weight (<4000 g vs. ≥ 4000 g).

^c^RR adjusted for pregnancy-related disease other than women with a suspected large-for-gestational-age fetus, cesarean during labor and birth weight (<4000 g vs. ≥ 4000 g).

Our study power, determined post hoc according to our results for our principal endpoint with α = 0.05, was 86% (one-sided test).

## Discussion

A policy of induction of labor between ≥ 37 weeks and ≤ 38 weeks^+ 6 days^ for women with a constitutionally LGA fetus among women without diabetes does not reduce perineal lesions.

Fetal weight is routinely estimated in France during the last fetal ultrasound, performed by a well-trained professional. French national health insurance reimburses three ultrasound examinations during low-risk pregnancies (one between 11 weeks^+0 d^ and 13 weeks^+6 d^, one between 20 and 24 weeks, and one between 30 and 35 weeks) in accordance with the national guidelines. This last ultrasound enables, among other things, the estimation of fetal weight. Fetal weight is generally assessed in France with the formula developed by Hadlock et al. [29].

In the studies looking at induction of labor for suspected fetal macrosomia, macrosomia is often defined as a fetal weight greater than 4000 g or 4500 g [19]. Few studies use a percentile cutoff for gestational age, as our work does [20]. The variation in the published results about neonatal and maternal morbidity can be explained in part by the variable definition of macrosomia — in utero or at birth. In our study we defined a LGA infant as a weight > 97^th^ percentile for gestational age, using growth curves that take the child’s sex into account [27]. These widely used French curves were constructed from 203,062 births. We chose the threshold > 97^th^ percentile to be symmetric with the definition of the international small-for-gestational age advisory board consensus statement of 2001 [30].

Another explanation for the variation in results among studies of induction of labor in macrosomia suspected in utero is the lack of contrast between the induction and control groups for gestational age at delivery [18]. We might think that induction of labor must be performed early enough to avoid excessive weight gain by the fetus and thus hope to diminish the risks of maternal or neonatal complications associated with macrosomia. It is for this reason that we compared, in an intention to treat analysis, women with inductions between ≥37 weeks and ≤38 weeks^+ 6 days^ with those who had expectant obstetrical management during this period (regardless of their management at or after 39 weeks).

Determining the optimal mode of delivery remains a topical research subject, particularly studies of the utility of inducing labor for fetuses with suspected macrosomia [18]. A policy of cesarean delivery was studied but appears inappropriate as it would increase the cesarean rate and would not be cost-effective [31,32]. For Kankins et al., “The range … for permanent brachial plexus injury that could be avoided with cesarean section on request would appear to vary between 1 in 5000 and 1 in 10,000 vaginal births” [33]. Moreover, we know that the risk of maternal mortality after a cesarean is higher than after vaginal delivery [34]. Known risks of cesarean deliveries include thromboembolic, infectious, traumatic, and hemorrhagic complications [35]. In the longer term, the risks of ectopic pregnancy, infertility, and placental problems are considerable [36]. The risk of a repeat cesarean also increases.

The studies concerning induction of labor in cases of macrosomia suspected in utero have paid little attention to perineal morbidity [19-22]. In our study, after adjustment, we found no statistically significant difference in the rates of either perineal lesions or episiotomies between the group of women whose labor was induced and the other group. This result is surprising because professionals know the risk of perineal lesions associated with the birth of a macrosomic child [37] and therefore should at least limit recourse to episiotomies in the group of women with inductions, since the point of the induction was to avoid the birth of a macrosomic child. We observed a lower rate of 3rd-degree perineal tears in the induction-of-labor group (0.8 vs. 1.4%) and, inversely, a higher rate of 4^th^ degree tears (1.6 vs. 0.1). These results must be interpreted cautiously, for we certainly lack the power to assess a potential reduction in the rate of severe perineal tears due to a policy of labor induction for LGA fetuses. Nonetheless, previous reports have observed that high fetal birth weight is associated with an elevated risk of anal sphincter damage to the mother [4,5].

Nor did we find any difference between our two groups for the rate of cesareans during labor. This result varies in different studies: some find a higher cesarean rate in cases of induction of macrosomic fetuses [17,20,22,38] while others do not [18,19,23]. A recent systematic review and meta-analysis concluded that the induction of labor in women with intact membranes for postdate or other indications (including 2 studies of suspected macrosomia and 1 of diabetes) reduces the risk of cesarean section (OR = 0.83; 95% CI:0.76-0.92) [39]. Furthermore, we found no increase in our study in the rate of immediate PPHs (>1 L). In a retrospective cohort study of an unselected population database, Stock et al. noted that elective induction of labor for no recognized medical indication at 37–41 weeks’ gestation compared with expectant management (continuation of pregnancy to either spontaneous labor, induction of labor, or cesarean section at a later gestational age, as in our study) was associated with a decreased odds of PPH [40]. The principal problem in this study is that the Scottish database did not record the indications for induction of labor so that unless maternal or fetal pathology was specifically mentioned, the induction of labor was considered to be elective.

Our study excluded women with diabetes because the macrosomic children of mothers with and without diabetes have different risks of neonatal injury [41].

A policy of induction of labor for LGA fetuses could be useful for reducing neonatal morbidity (brachial plexus injury, etc.). The results of the randomized trial with the largest number of subjects thus far published found, contrary to our results, a reduction in neonatal traumas (defined as shoulder dystocia, fracture of the clavicle and brachial plexus injury, or perinatal death) among the induction group: RR = 0.34 (95% CI: 0.16-0.71) [23]. We note that our study lacks the power to show an effect of induction of labor on perinatal morbidity and mortality.

The literature has long underlined the neonatal and maternal complications associated with the birth of a child with macrosomia [10-15]. The correlation between rates of maternal and neonatal morbidity increases with the extent of in utero macrosomia (<4000 g, 4000–4499 g, ≥ 4500 g) [39]. The proportion of composite morbidity in these three groups was: 26.2%, 41.2%, and 63.6% (p < 0.0001) [42]. It is important to optimize the identification of LGA fetuses in utero at term if one hopes to prevent maternal or neonatal complications by recommending a policy of induced labor before 39 weeks and to avoid futile medicalization of a pregnancy for which fetal weight was overestimated. These misestimates of fetal weight can result in greater recourse to cesareans [43,44]. Moreover, the methods for estimating fetal weight, especially macrosomia, remain unreliable [45,46]. Consequently no particular type of ascertainment (clinical or ultrasound) of macrosomic fetuses can be privileged. Our study shows that only 26.94% of the fetuses identified as LGA in utero were found to be LGA at birth; inversely 2.24% of fetuses not suspected in utero were LGA at birth (Figure 1). A study seeking to identify fetuses ≥4500 g (based on palpation and fundal height measurement) at admission to the labor room found the sensitivity of the clinical examiner was 43% and the specificity 99.8% (macrosomia was suspected in 19 of 4480 deliveries) [47]. A Bayesian calculation indicates that the post-test probability of detecting a macrosomic fetus (>4000 g) in an uncomplicated pregnancy varies from 15% to 79% according to sonographic estimates of birth weight, and from 40 to 52% with clinical estimates [46].

One limitation of this study may be the absence in our database of reliable data about the mode of dating pregnancies: this information is missing for many women. Nonetheless, in France, as we pointed out above, the national health insurance fund reimburses three ultrasound examinations during pregnancy. In 2003 and 2010, the national perinatal survey found only 0.1% and 0.2% of women who had had no ultrasound during pregnancy [48]. It is therefore unlikely that our results are biased (weight being correlated with term at birth) by the absence of data about an early fetal ultrasound. The second limitation of this study is that we could not include in our analysis the maternal weight gain during pregnancy and any prior history of macrosomia because of the high rate of missing data. The third limitation is its long study period, between 1994 and 2008. Although the skills of French sonographers have probably been at a consistent level since 1994, the quality of the ultrasound equipment used has undoubtedly varied, in view of technological advances over this period. To take this variety into account in our analysis, we examined results by year of delivery (in 3 four-year categories). The last limitation of our work is its retrospective cohort design, more susceptible to bias than prospective cohort studies or randomized studies. However, the elements likely to induce such bias, such as knowledge of outcome at exposure measurement, recall bias, and loss to follow-up, are absent from our historical cohort intention-to-treat study in which fetal weight was estimated and mode of planned delivery determined during the pregnancy and before delivery. In particular, the exposure information was collected before the outcome.

## Conclusions

In utero identification of constitutionally LGA fetuses among non-diabetic women would not be useful in reducing maternal morbidity. This result reinforces our earlier work which found that in utero identification of babies born with macrosomia (≥4000 g) did not improve maternal outcomes [49].

## Competing interests

The authors declare that they have no competing interest.

## Authors’ contributions

FV conceived the study and drafted the manuscript. FV and OR conducted the data analysis. All authors contributed to the interpretation of the results with their critical comments and to the drafting of the manuscript. All authors have read and approved the final manuscript.

## Authors’ information

FV, MD, PhD, is an associate professor in epidemiology and an obstetrician-gynecologist.

OR, Mr is a statistician.

BN, Ms, is a midwife supervisor.

DL, MD, PhD, is a professor in obstetrics and gynecology.

## Pre-publication history

The pre-publication history for this paper can be accessed here:

http://www.biomedcentral.com/1471-2393/14/156/prepub
